# Does Dyadic Coping Predict Couples’ Postpartum Psychosocial Adjustment? A Dyadic Longitudinal Study

**DOI:** 10.3389/fpsyg.2020.561091

**Published:** 2020-09-25

**Authors:** Stephanie Alves, Ana Fonseca, Maria Cristina Canavarro, Marco Pereira

**Affiliations:** Center for Research in Neuropsychology and Cognitive and Behavioral Intervention (CINEICC), Faculty of Psychology and Educational Sciences, University of Coimbra, Coimbra, Portugal

**Keywords:** dyadic coping, psychosocial adjustment, transition to parenthood, actor–partner effects, longitudinal

## Abstract

The way couples jointly manage pregnancy-related demands may prevent both members from experiencing psychosocial maladjustment after childbirth. This study examined (a) changes in dyadic coping (DC) and indicators of psychosocial adjustment [depressive and anxiety symptoms and quality of life (QoL)] from the second trimester of pregnancy (T1) to 6 weeks postpartum (T2), (b) the actor and partner effects of DC at T1 on couples’ adjustment at T2, and (c) whether changes in DC over time would be associated with changes in the adjustment of both women and their partners. This study adopted a prospective quantitative dyadic longitudinal design. A total of 303 couples from Portugal answered self-report questionnaires assessing DC, depressive and anxiety symptoms, and QoL at T1, of which 290 were contacted at T2 to complete the same measures (*n* = 138 couples returned the questionnaires). Results showed that first-time fathers’ QoL and both first and experienced fathers’ stress communication decreased over time, as did common DC (i.e., the way couples cope together with stress) perceived by both partners. First-time mothers reported higher increases in negative DC. The more positive DC the women provided to men at T1, the higher the internalizing symptoms of women at T2; the more the women communicated stress at T1, the higher the internalizing symptoms of men at T2. Both partners’ common DC at T1 positively predicted their QoL at T2. The larger the decrease in common DC over time, the greater the increase in internalizing symptoms of couples and the greater the decrease in their QoL. These findings suggest that DC strategies should be considered into the psychosocial care of couples becoming parents, as a relevant coping resource that partners could use to help each other in situations of stress. More than (exclusively) encouraging the men’s role as support providers, couples should be encouraged to reserve time for one another, to discuss each other’s concerns, and to seek for solutions as a team. These strategies should be promoted before, and fostered after, childbirth. Likewise, clinicians should be aware that partners might not feel equally comfortable with specific DC strategies and then should be carefully addressed among couples.

## Introduction

The birth of a child leads to several readjustments in the familial system, which couples may experience as stressful and challenging (Cowan and Cowan, 2000). In fact, because expecting/having a child affects both members of a couple at the same time and concerns them as a unit (McGoldrick and Carter, 2003), this period may be conceptualized as a context of dyadic stress (Bodenmann, 2005), during which both partners need to cope not only with one’s own stress but also with the other’s needs and shared concerns within the couple (Bodenmann et al., 2016, 2017).

Unsuccessful coping efforts may impair couples’ psychosocial adjustment. High levels of depressive symptoms affect between 4.1 and 15.6% of men prenatally and between 2.4% and 41.2% of men postnatally (Cameron et al., 2016), whereas the prevalence of anxiety symptoms is estimated to range from 4.1 to 16.0% during pregnancy and 2.4–18.0% after childbirth (Leach et al., 2016). Despite potential increases in depressive symptoms and decreases in anxiety symptoms, there is overall stability in men’s symptoms over the prenatal and postnatal periods (Cameron et al., 2016; Leach et al., 2016), whereas the prevalence of depressive and anxiety symptoms among women is estimated to be relatively higher during pregnancy (17 and 23%, respectively) than after childbirth (13 and 15%, respectively) (Underwood et al., 2016; Dennis et al., 2017). In addition, a decline in quality of life (QoL) has been found to be common after childbirth; however, relatively few studies have explored its course across the transition to parenthood, with mixed findings being reported (e.g., Condon et al., 2004; Pilkington et al., 2015; Chen et al., 2019). Accordingly, it is important to improve our understanding of which dyadic resources, such as engagement in dyadic coping (DC), should be promoted early to help both women and their partners successfully adapt after childbirth.

The systemic–transactional model (STM; Bodenmann, 2005) conceptualizes stress experiences and coping from a “we stress” perspective, highlighting the interdependence and mutuality between members of a couple (i.e., stressors always directly or indirectly affect both partners in a committed relationship, and the resources of one partner expand the resources of the other) (Bodenmann et al., 2016, 2017). According to this framework, DC is as a process that is triggered when stress is communicated (either verbally or non-verbally) by one partner and decoded/interpreted by the other partner (or by both partners when dealing with a shared stressor). DC covers distinct forms of reactions that are grouped into positive and negative. Positive reactions include supportive DC (e.g., one partner helps with daily tasks, provides advice, helps reframe the situation, or expresses empathic understanding and solidarity), delegated DC (i.e., one partner takes over tasks at the demand of the other partner to alleviate his/her stress), and common DC (i.e., both partners cope with common stressors by engaging in joint coping efforts, such as joint problem solving and information seeking, or sharing of feelings). Examples of negative DC behaviors are when one partner provides support by minimizing the other’s stress or using sarcasm or open disinterest (hostile reactions), when one partner provides support unwillingly and with no motivation (ambivalent reactions), or when one partner provides support without real motivation (superficial reactions) (Bodenmann, 2005).

Dyadic coping is an interrelated but distinct concept from general partner support, which has been widely examined in the perinatal literature; indeed, whereas research focused on QoL has mostly addressed the influence of broad social support, making it difficult to separate the specific role of the partner (e.g., Webster et al., 2011), the associations between partner support and couples’ depressive and anxiety symptoms have been largely documented (for a review see Pilkington et al., 2015). However, those studies have privileged an individual perspective (mostly taken into account the woman’s perception of the couple’s characteristics and her adjustment) and mostly adopted a cross-sectional design, thereby limiting inferences about the truly protective role of partner support in the long term. In addition, the term “partner support” has been unclearly defined across studies as well as examined within the broader context of protective factors (rather than as the central topic), which therefore makes the translation of current evidence into concrete intervention strategies difficult (Pilkington et al., 2015; Mickelson and Biehle, 2017).

This is the most distinctive feature from DC, as DC is anchored in a robust model of interpersonal coping (the STM) with large empirical evidence and focuses on the experience of stress and coping in couples (rather than general partner support such as helping with household), more specifically on how external stressors (e.g., childcare demands, work–family conflicts, potential disagreements with family of origin) directly or indirectly impact both partners and how couples may cope with them together to avoid tensions and arguments within the couple (internal stress). Also, it includes other forms of supportive processes (e.g., joint coping efforts) in addition to the support provided by one partner to the other (i.e., supportive behaviors) (Bodenmann and Randall, 2012), as usually operationalized in the perinatal literature.

Notwithstanding the contributions of existing research, at least two specificities of the transition to parenthood highlight the need to go beyond the broader coping and support literature in this area and address DC components. First, this is a period characterized by great expression of needs and requests for support, particularly by women (Levy-Shiff, 1999; Cowan and Cowan, 2000); thus, the unique effects of stress communication underlying the activation of DC behaviors should be better understood. This is especially relevant given the fact that interpersonal relationship skills (e.g., skills to communicate effectively and to ask for help in time of need) may contribute more for couples’ adjustment to the birth of a child than their social network (Ketner et al., 2018). Second, several stressors of this period are likely to be appraised as concerning both members of the couple (i.e., transition to parenthood as a “we stress” period). In fact, even when partners experience personal concerns at some point (e.g., physical changes during pregnancy, work–family conflict), these can have a serious impact on the other and the couple as a whole (i.e., crossover effects within the couple; Westman, 2011). Accordingly, both partners’ coping efforts are triggered not only to respond to the other’s needs (i.e., partner-oriented behaviors) but also to promote one another’s individual and relational well-being (i.e., couple-oriented behaviors) (Bodenmann, 2005). Disentangling the contribution of distinct DC strategies will help identify accurate prevention targets for couple-based interventions.

The literature on DC during the transition to parenthood is relatively recent and yields initial evidence of the associations between DC and dyadic adjustment (Molgora et al., 2019; Brandão et al., 2020), depressive symptoms (Alves et al., 2018), and QoL (Brandão et al., 2020) during pregnancy. Recently, two prospective longitudinal studies also showed that common DC and perceived similarity in DC within the couple influenced partners’ individual and parental adjustment after childbirth (Alves et al., 2019, 2020). Stress communication and positive DC strategies have been found to be negatively associated with depressive symptoms (Rottmann et al., 2015) and positively associated with QoL in couples experiencing several health conditions (Meier et al., 2011; Vaske et al., 2015; Ernst et al., 2017). Conversely, negative DC behaviors have been found to be associated with increased psychological distress (Rottmann et al., 2015) and poor QoL (Meier et al., 2011; Vaske et al., 2015). Moreover, these studies have demonstrated that, consistent with the APIM (Kenny et al., 2006), one partner’s DC influences not only his/her own adjustment (actor effects) but also his/her partner’s adjustment (partner effects).

Although the transition to parenthood is a normative life transition, similar to the experience of dealing with one partner’s serious health problem, this period is likely to be experienced as “we stress” (Bodenmann et al., 2016, 2017). Additionally, the adjustment process to the birth of a child may be marked by emotional (as previously described) and marital (Delicate et al., 2018) strains, as it seems to be the case in the context of chronic illness (Meier et al., 2011; Rottmann et al., 2015). Therefore, because DC influences couples’ adjustment to shared and potentially stressful events, the way that couples prenatally engage in DC strategies is likely to impact their adjustment to the birth of a child.

The results of these studies also elucidate that the adaptiveness of certain DC strategies may be dependent, for example, on the different roles of each member within the couple (e.g., patient vs. caregiver; Rottmann et al., 2015; Ernst et al., 2017). The traditional roles assumed by women (as the principal caregivers of the child) and men (as the breadwinners) during the transition to parenthood (Katz-Wise et al., 2010) have been challenged by the increasing changes in family life over the past years (e.g., greater involvement of fathers in childcare; Cabrera et al., 2018). For instance, although the Portuguese cultural context strongly endorses traditional gender roles (Aboim, 2010), there is a dominant configuration of full-time dual-earner parents and a changing conception of fatherhood in Portugal (Escobedo and Wall, 2015; Wall and Leitão, 2017). Accordingly, this could lead to a new understanding of the transition to parenthood, which, contrary to previous studies (Levy-Shiff, 1999), may translate into more similarities than differences between women’s and men’s support needs in times of stress.

## The Present Study

The aims of the present study were to (a) assess changes in indicators of individual adjustment (depressive and anxiety symptoms and QoL) and forms of DC from the second trimester of pregnancy (time 1, T1) to 6 weeks postpartum (time 2, T2) in both women and men; (b) examine the effects of DC (assessed at T1) on both women and their partners’ psychosocial adjustment at T2; and (c) explore whether changes in DC over time would be associated with changes in both women and their partners’ adjustment. Because having prior children versus experiencing first-time parenthood may influence DC requests, we controlled for parity in all analyses to ensure that the effects of DC on couples’ adjustment were not due to this variable. We established the following hypotheses. First, we expected that women’ levels of depressive and anxiety symptoms would decrease (hypothesis 1a), whereas men’ levels of symptoms would remain stable from T1 to T2 (hypothesis 1b). Given the few and mixed results observed for QoL, we did not establish hypotheses regarding this outcome. Likewise, we adopted an exploratory approach regarding the course of DC over time. Second, we expected that higher levels of stress communication and positive and common DC would predict less internalizing symptoms and more QoL and that higher levels of negative DC would predict more internalizing symptoms and less QoL (hypothesis 2). In addition, we expected that one partner’s DC would predict not only their own (hypothesis 2a) but also the other partner’s adjustment as well (hypothesis 2b). Finally, we hypothesized that decreases in positive forms of DC would be associated with increases in internalizing symptoms and decreases in QoL over time, whereas the inverse relationships were expected for negative DC (hypothesis 3).

## Materials and Methods

### Participants

The sample consisted of 303 heterosexual couples recruited during the second trimester of pregnancy (gestational weeks, mean = 23.00, *SD* = 5.30; range = 12–37). Sixty-two percent were married couples living together, and 34.3% were unmarried couples cohabitating (relationship length, mean = 7.16 years, *SD* = 4.49). The majority were expecting their first child (60.7%). Compared with men, women were younger [women: age, mean = 31.61, *SD* = 4.66; men: age, mean = 33.74, *SD* = 5.15; *t*_(__300__)_ = -9.07, *p* < 0.001, *d* = 0.61] were more likely to have university education [61.5% vs. 41.8%; χ^2^(2) = 50.45, *p* < 0.001, φ_c_ = 0.29] and reported being employed with significantly less frequency [84.0% vs. 93.0%; χ^2^(1) = 11.74, *p* = 0.001, φ_c_ = 0.14]. Regarding prior history of psychopathology, a high proportion of women reported previous psychological problems [34.4% vs. 5.5%; χ^2^(1) = 77.09, *p* < 0.001, φ_c_ = 0.36] and psychological treatment [27.3% vs. 9.5%; χ^2^(1) = 31.36, *p* < 0.001, φ_c_ = 0.23]. A history of pregnancy loss was reported by 18.5% of women and a history of infertility by 10.6% of women. Most women had a planned (77.6%) and desired (97.0%) pregnancy, which occurred without gestational complications (65.0%).

### Procedure

This study was approved by the Research Ethics Committees of the Faculty of Psychology and Educational Sciences of the University of Coimbra and one university hospital (Centro Hospitalar e Universitário de Coimbra, EPE). The inclusion criteria were as follows: (1) women were in the course of the second trimester of a singleton pregnancy, without any major complications with the baby (e.g., fetal anomalies) or other adverse clinical events (e.g., perinatal loss); (2) the partners were in a relationship (formally married, cohabiting or dating); (3) both partners were at least 18 years old; and (4) both partners were able to read and understand Portuguese.

From November 2015 to May 2017, eligible women (and their partners, if available) followed in the Maternity Daniel de Matos were informed about the study by their obstetrician. Those who agreed to be contacted by the researchers were presented the study aims and invited to participate (consecutive sampling). A signed consent form was obtained from all participants, and a copy was given to each member of the couple. At this time (second trimester of pregnancy—T1), each member of the couple received a set of questionnaires and was asked to complete them separately at home and return them in a sealed envelope at the next obstetric appointment. We focused specifically on the second trimester of pregnancy because this is a relatively stable trimester in terms of emotional adjustment (Figueiredo and Conde, 2011; Cameron et al., 2016), during which both partners become more aware of the baby’s reality (Canavarro, 2001; Kowlessar et al., 2015). At 6 weeks postpartum (T2), couples were mailed two versions of the questionnaires (one for each partner) along with a prestamped envelope in which to return them after completion. At T1, a text message was sent to all couples 1 or 2 days before the appointment to remind couples to bring the completed questionnaires to the appointment. At T2, the researchers sent out one reminder after 2 weeks.

A total of 611 women (or couples, when applicable) were initially contacted at T1; 52 of these couples declined to participate, and eight did not meet the inclusion criteria at the time of the study’s presentation. Of the 551 couples who agreed to participate, 335 returned questionnaires (participation rate = 60.8%), 32 of whom were excluded because the questionnaires were filled out only by the woman (*n* = 25) or showed, at T2, that they no longer met the criteria for participation. At T2, 290 of the 303 couples who were retained at T1 were mailed questionnaires (five couples were not contacted because of perinatal loss and eight because of the absence of delivery information); 138 of these couples returned questionnaires that were answered by both partners (participation rate = 47.6%). On average, couples returned the T2 questionnaires when their children were between 6 and 11 weeks (82.7%; mean = 9.40, *SD* = 3.12, range = 6–21).

The differences between couples who completed the assessment at both times and those who dropped out were assessed regarding sociodemographic and obstetric data as well as baseline individual adjustment. Men from couples who participated at both assessment times were more likely to have completed high school than those who were contacted but dropped out at T2, χ^2^(2) = 8.79, *p* = 0.012, φ_c_ = 0.18. Women who were retained at T1 and T2 were more likely to have a university education, χ^2^(2) = 6.71, *p* = 0.035, φ_c_ = 0.15, and a planned pregnancy, χ^2^(1) = 4.60, *p* = 0.032, φ_c_ = 0.13, than those who only participated at T1. No significant differences were found in the remaining variables. The analyses were run using the 303 couples who completed the T1 assessment.

### Measures

#### Internalizing Symptoms

Depressive symptoms were assessed with the Edinburgh Postnatal Depression Scale (Cox et al., 1987). Participants should respond to 10 items on a 4-point response scale considering the last 7 days. A total score is obtained ranging from 0 to 30. Higher values reflect higher levels of depressive symptoms. Cronbach’s α values for the present sample were 0.86 for women and 0.83 for men at T1 and 0.83 for women and 0.81 for men at T2. Anxiety symptoms were assessed using the Anxiety subscale (seven items) of the Hospital Anxiety and Depression Scale (HADS; Zigmond and Snaith, 1983). Each item is answered on a 4-point scale, considering the last week. The total score ranges between 0 and 21. Higher scores denote higher levels of anxiety symptoms. Cronbach’s α values for this study were 0.84 for women and 0.78 for men at T1 and 0.79 for women and 0.81 for men at T2. Because depressive and anxiety symptoms scores were reliably correlated (*r* > 0.70, *p* < 0.001) in both women and men and at each assessment point, the scores were averaged to create an aggregate measure of internalizing symptoms.

#### Quality of Life (QoL)

Quality of life was assessed using the EUROHIS-QOL 8-Index (Power, 2003), which consists of eight items (two for each domain of QoL—physical, psychological, social relationships, and environment) that are answered on 5-point response scales (e.g., from “not at all” to “completely,” from “very dissatisfied” to “very satisfied”) considering the previous 2 weeks. A global score is obtained from the sum of all items, with higher scores indicating a better perception of QoL. In this study, Cronbach’s α’s were 0.76 for women and 0.80 for men at T1 and 0.78 for women and 0.85 for men at T2.

#### Dyadic Coping (DC)

Distinct strategies of DC were assessed using the five subscales of the Dyadic Coping Inventory (Bodenmann, 2008), assessing own stress communication (four items; e.g., “I ask my partner to do things for me when I have too much to do”), own supportive DC (five items; e.g., “I show empathy and understanding to my partner”), own delegated DC (two items; e.g., “When my partner feels he/she has too much to do, I help him/her out”), own negative DC (four items; e.g., “When my partner is stressed I tend to withdraw”), and common DC (five items; e.g., “We try to cope with the problem together and search for ascertained solutions”). Each item was rated on a 5-point scale (1 = “very rarely” to 5 = “very often”), and a total score for each subscale was calculated by computing the mean of the respective items. Higher scores indicate more of the behavior of interest. For simplicity, the two subscale scores of supportive and delegated DC were combined to yield an index of positive DC. In our sample, Cronbach’s α ranged from 0.67 (stress communication – women) to 0.89 (common DC—women) at T1 and from 0.73 (stress communication—women) to 0.91 (common DC—women) at T2.

### Data Analysis

Descriptive statistics were performed for sample characterization in SPSS (IBM SPSS, version 23.0), and χ^2^ tests and paired *t*-tests were conducted to assess the differences between women and men. Descriptive statistics for and correlations between the main study variables at T1 and T2 were also computed. Parity was included as a covariate in all analyses, as well as the timing of pregnancy assessment and the timing of postpartum assessment in order to control for the considerable heterogeneity regarding compliance with the assessment schedule across participants. To improve clarity, covariates were only reported in the “*Results*” section if significant.

Univariate LCS (McArdle, 2009) models were computed in Mplus, version 8 (Muthén and Muthén, 1998–2017), to examine changes over time in each variable. Change between T1 and T2 was modeled as a latent factor, which allowed us to estimate the mean/intercept of the change (μ_Δ_; the average change over time) and the variance/residual variance of the change (σ^2^_Δ_; the extent to which individuals differ in the change they manifest over time). A significant positive mean/intercept of the LCS factor indicates, on average, an increase for individuals over time, and a significant negative mean/intercept suggests a decrease for individuals over time; a significant variance/residual variance in the LCS factor indicates there is heterogeneity across individuals regarding the average trajectory (McArdle, 2009; Henk and Castro-Schilo, 2016).

To assess the role of DC strategies in women’s and their partners’ individual adjustment, we conducted APIMs in Mplus. This approach accounts for the interdependence of women’s and men’s scores within dyads by specifying correlations between all of the predictor variables and between the error disturbances for the two outcome variables. An APIM was separately computed for each of the two indicators of individual adjustment assessed at T2 (internalizing symptoms and QoL), considering the four DC subscales assessed at T1 as independent variables (stress communication, positive DC, negative DC and common DC, for each partner) and controlling for parity, timing of pregnancy assessment, and timing of postpartum assessment, as well as for the baseline level of the respective outcome for each partner. Accordingly, each APIM included eight predictors and five covariates. Within the same model, it allows estimating actor (i.e., the degree to which a person’s own DC predicts that person’s individual adjustment) and partner (i.e., the degree to which a person’s partner’s DC predicts that person’s individual adjustment) effects for both members of the couple. All predictors were centered around the grand mean and unstandardized path coefficients, and their standard errors were reported (Kenny et al., 2006).

Finally, to examine whether changes in DC subscales were related to changes in individual adjustment over time within and across partners, we conducted APIMS using two-wave LCS models (2W-LCS; Henk and Castro-Schilo, 2016). This approach has been recently proposed to examine change-to-change effects with two-wave data; briefly, it provides estimates for the relationship among LCS factors. To increase interpretability of the means of the LCS factors, we used the original scores instead of the mean-centered scores, and regression coefficients were interpreted as with any linear regression (e.g., a positive regression coefficient indicates that higher/lower change scores in a variable are associated with higher/lower change scores in the other variable). The terms “higher” and “lower” should be substituted by “increases” and “decreases,” respectively, when the mean of the LCS is significant (Henk and Castro-Schilo, 2016). Beyond considering the χ^2^ statistic—which needs to be statistically non-significant (*p* > 0.05) to indicate good model fit but is highly sensitive to large sample sizes (Marôco, 2010)—we assessed the models’ fit based on additional criteria: a comparative fit index (CFI) > 0.95, a root mean square error of approximation (RMSEA) < 0.05, and a standardized root mean square residual (SRMR) < 0.08 (Hu and Bentler, 1998). We added auxiliary variables (i.e., those variables that directly influence missingness: education and a planned pregnancy) in all models following Graham (2003) recommendations, in order to minimize bias and enhance power (Graham, 2003; Enders, 2010).

Effect sizes were interpreted as follows: small: *d* ≥ 0.20, φ_c_ ≥ 0.10, *r* ≥ 0.10, *R*^2^ ≥ 0.02; medium: *d* ≥ 0.50, φ_c_ ≥ 0.30, *r* ≥ 0.30, *R*^2^ ≥ 0.13; large: *d* ≥ 0.80, φ_c_ ≥ 0.50, *r* ≥ 0.50, *R*^2^ ≥ 0.26 (Cohen, 1988). Significance was set at the level *p* < 0.05. Missing data were handled using FIML in Mplus, an approach that uses all data available to estimate models (Enders and Bandalos, 2001).

## Results

### Individual Adjustment and DC in Women and Their Partners Over Time

**FIGURE 1:**
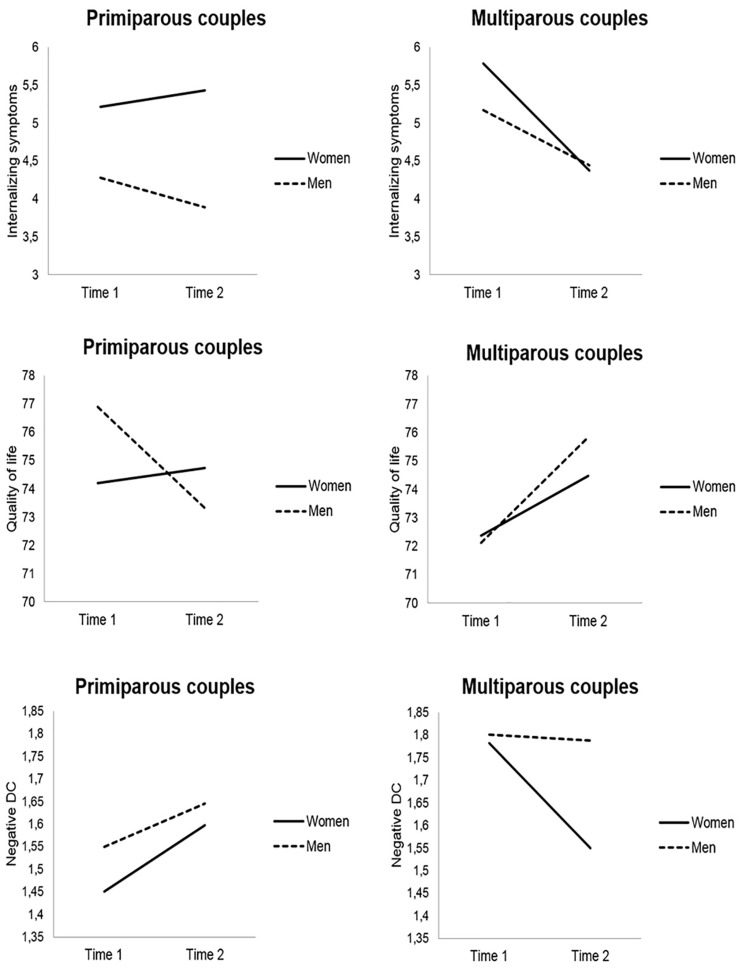
Mean scores of internalizing symptoms, quality of life, and negative dyadic coping (DC) by parity (primiparous vs. multiparous couples) and time (time 1 = second trimester of pregnancy; time 2 = 6 weeks postpartum), adjusted for timing of pregnancy assessment and timing of postpartum assessment. Only the variables for which parity significantly predicted latent change scores are illustrated.

As presented in Table 1, on average, women’s engagement in common DC decreased and their negative DC increased over time. Parity was significantly associated with the LCS of negative DC (*B* = −0.33, *p* < 0.001), indicating that first-time mothers reported higher increases in negative DC (Figure 1). A significant reduction in stress communication and common DC over time was observed among men. Men showed significant decreases in QoL over time, but this change was conditional on parity (*B* = 6.02, *p* = 0.001); the positive coefficient and Figure 1 indicate that first-time fathers reported higher decreases in QoL from T1 to T2. For both women and men, the intercept of the LCS for internalizing symptoms was statistically significant before accounting for the influence of parity (and the other covariates), suggesting that this variable somewhat influenced the trajectory of internalizing symptoms. For women, parity was significantly associated with the LCS of internalizing symptoms (*B* = −1.46, *p* = 0.001), suggesting that there were lower change scores for (or a trend toward decreases in) internalizing symptoms for multiparous women (Figure 1).

**TABLE 1:** Individual adjustment and dyadic coping: descriptive statistics and univariate LCS models.

	**Time 1**		**Time 2**		**Time differences**			
	**Women**	**Men**	**Women**	**Men**	**Women**		**Men**	
	**Mean (*SD*)**	**Mean (*SD*)**	**Mean (*SD*)**	**Mean (*SD*)**	**μ_Δ_**	**σ^2^_Δ_**	**μ_Δ_**	**σ^2^_Δ_**
IS	5.62 (3.73)	4.50 (3.21)	5.04 (3.14)	4.10 (3.01)	−0.01	8.24***	−0.42	5.58***
QOL	72.91 (11.31)	75.72 (11.75)	74.75 (11.60)	74.15 (11.98)	0.60	100.92***	−3.36**	111.81***
SC	4.03 (0.60)	3.59 (0.66)	4.00 (0.65)	3.48 (0.75)	−0.10	0.38***	−0.18*	0.45***
PDC	3.97 (0.56)	3.92 (0.53)	3.96 (0.59)	3.88 (0.59)	−0.05	0.28***	−0.04	0.23***
NDC	1.61 (0.59)	1.72 (0.65)	1.57 (0.59)	1.69 (0.63)	0.12*	0.30***	0.04	0.35***
CDC	3.95 (0.75)	3.90 (0.69)	3.89 (0.83)	3.81 (0.74)	−0.16*	0.47***	−0.21***	0.32***

LCS, latent change score; time 1, second trimester of pregnancy; time 2, 6 weeks postpartum; IS, internalizing symptoms (scores range = 0–25,5); QOL, quality of life (scores range = 0–100); SC, stress communication (scores range = 1–5); PDC, positive dyadic coping (DC) (scores range = 1–5); NDC, negative DC (scores range = 1–5); CDC, common DC (scores range = 1–5); μ_Δ_, intercept of the latent change factor; σ^2^_Δ_, residual variance of the latent change factor. Women’s education and planned pregnancy were included as auxiliary variables in the LCS models conducted for women and men’s education in the LCS models conducted for men. The unstandardized estimates for μ_Δ_ and σ^2^_Δ_ are adjusted for covariates. *p < 0.05, **p < 0.01, ***p < 0.001.

The correlations between study variables are shown in Table 2. Correlations within dyads suggest non-independence between partners’ data and thus support the relevance of adopting a dyadic approach—the APIM—that allows incorporating both actor and partner effects.

**TABLE 2:** Intercorrelations between study variables at T1 and T2.

	**1**	**2**	**3**	**4**	**5**	**6**	**7**	**8**	**9**	**10**	**11**	**12**
(1) SC (T1)	0.14*	0.55***	0.37***	0.42***	−0.36***	−0.42***	0.43***	0.45***	−0.14*	−0.19*	0.21***	0.25**
(2) SC (T2)	0.49***	0.35***	0.32***	0.59***	−0.39***	−0.43***	0.39***	0.64***	−0.31***	−0.36***	0.23**	0.37***
(3) PDC (T1)	0.39***	0.34***	0.46***	0.63***	−0.45***	−0.57***	0.64***	0.46***	−0.26***	−0.28**	0.27***	0.21*
(4) PDC (T2)	0.29***	0.58***	0.59***	0.44***	−0.46***	−0.66***	0.63***	0.75***	−0.43***	−0.40***	0.42***	0.40***
(5) NDC (T1)	−0.24***	−0.26**	−0.44***	−0.35***	0.42***	0.55***	−0.49***	−0.45***	0.36***	0.37***	−0.22***	−0.29**
(6) NDC (T2)	−0.17*	−0.37***	−0.36***	−0.52***	0.52***	0.44***	−0.61***	−0.64***	0.48***	0.36***	−0.36***	−0.31***
(7) CDC (T1)	0.31***	0.31***	0.68***	0.57***	−0.40***	−0.39***	0.59***	0.67***	−0.24***	−0.37***	0.25***	0.42***
(8) CDC (T2)	0.27**	0.55***	0.53***	0.75***	−0.34***	−0.53***	0.63***	0.52***	−0.44***	−0.49***	0.31***	0.53***
(9) IS (T1)	−0.11*	–0.07	−0.27***	−0.24**	0.29***	0.20*	−0.25***	−0.21*	0.37***	0.71***	−0.52***	−0.49***
(10) IS (T2)	0.05	–0.12	–0.01	−0.27**	0.02	0.23**	–0.06	−0.32***	0.60***	0.31***	−0.44***	−0.59***
(11) QOL (T1)	0.14*	0.12	0.34***	0.34***	−0.27***	−0.22**	0.36***	0.29**	−0.64***	−0.34***	0.27***	0.55***
(12) QOL (T2)	0.14	0.23**	0.25**	0.37***	–0.12	–0.12	0.24**	0.37***	−0.50***	−0.53***	0.61***	0.19*

Correlations for women are presented below the diagonal, and for men above the diagonal. Correlations within couples are shown in bold on the diagonal. T1, time; T2, time 2; SC, stress communication; PDC, positive dyadic coping (DC); NDC, negative DC; CDC, common DC; IS, internalizing symptoms; QOL, quality of life. *p < 0.05, **p < 0.01, ***p < 0.001.

### Actor and Partner Effects of DC at Pregnancy on Postpartum Individual Adjustment

The selection of the model included preliminary steps. Because we did not expect differences between women and men, we first constrained all the actor effects and partner effects, respectively, to be equal across gender, and we assessed the model’s fit of these constrained models. We obtained a significant χ^2^ test statistic (*p* < 0.05) for the model of internalizing symptoms [internalizing symptoms: χ^2^(13) = 33.09, *p* = 0.002; QoL: χ^2^(13) = 16.61, *p* = 0.218]. To identify model misspecification, we examined the modification index (MI) in combination with the expected parameter change, as recommended by Saris et al. (1987). Accordingly, we gradually unconstrained the parameters and observed a change in the model fit (χ^2^ difference test for nested models; Δχ^2^). All the paths could be equalized across gender without significant declines in the model fit, except the effects of prior children (Δχ^2^ = 7.65, Δ*df* = 1, *p* = 0.006), the actor effects of positive DC (Δχ^2^ = 9.44, Δ*df* = 1, *p* = 0.002), and the partner effects of stress communication (Δχ^2^ = 8.24, Δ*df* = 1, *p* = 0.004), which were left to vary freely between women and men. The final models fitted the data well (internalizing symptoms: χ^2^ = 11.84, *df* = 10, *p* = 0.296; RMSEA = 0.025; SRMR = 0.019; CFI = 0.998; QoL: χ^2^ = 16.61, *df* = 13, *p* = 0.218; RMSEA = 0.030; SRMR = 0.029; CFI = 0.996) and explained a high proportion of variance in the outcomes (Table 3).

**TABLE 3:** Effects of dyadic coping at pregnancy (T1) on women and their Partners’ individual adjustment at postpartum (T2).

	**Women**				**Men**			
	**IS**		**QOL**		**IS**		**QOL**	
	***R*^2^ = 0.50**		***R*^2^ = 0.39**		***R*^2^ = 0.49**		***R*^2^ = 0.39**	
	***B* (*SE*)**	***p***	***B* (*SE*)**	***p***	***B (SE)***	***p***	***B (SE)***	***p***
**Women**								
Baseline score of the outcome	0.60 (0.05)	<0.001	0.58 (0.06)	<0.001	0.06 (0.05)	0.217	0.03 (0.06)	0.620
Stress communication	0.32 (0.26)	0.223	1.42 (1.08)	0.187	**0.77 (0.35)**	**0.026**	0.69 (1.07)	0.519
Positive dyadic coping	**1.05 (0.45)**	**0.019**	−2.72 (1.57)	0.084	−0.33 (0.36)	0.358	2.49 (1.55)	0.108
Negative dyadic coping	0.03 (0.29)	0.909	0.88 (1.20)	0.464	−0.27 (0.29)	0.338	1.70 (1.19)	0.153
Common dyadic coping	−0.26 (0.32)	0.430	**3.64 (1.38)**	**0.009**	0.17 (0.32)	0.604	−1.44 (1.39)	0.299
**Men**								
Baseline score of the outcome	0.06 (0.05)	0.217	0.03 (0.06)	0.620	0.60 (0.05)	<0.001	0.58 (0.06)	<0.001
Stress communication	−0.60 (0.35)	0.092	0.69 (1.07)	0.519	0.32 (0.26)	0.223	1.42 (1.08)	0.187
Positive dyadic coping	−0.33 (0.36)	0.358	2.49 (1.55)	0.108	−0.50 (0.43)	0.252	−2.72 (1.57)	0.084
Negative dyadic coping	−0.27 (0.29)	0.338	1.70 (1.19)	0.153	0.03 (0.29)	0.909	0.88 (1.20)	0.464
Common dyadic coping	0.17 (0.32)	0.604	−1.44 (1.39)	0.299	−0.26 (0.32)	0.430	**3.64 (1.38)**	**0.009**
Parity^a^	−**1.33 (0.43)**	**0.002**	**3.27 (1.27)**	**0.010**	0.12 (0.40)	0.771	**3.27 (1.27)**	**0.010**

*Unstandardized maximum likelihood estimates are described. Significant estimates are in bold. ^a^0 = primiparous couples; 1 = multiparous couples. IS, internalizing symptoms; QOL, quality of life.*

Women’s positive DC at T1 significantly and positively predicted their own internalizing symptoms at T2. Women with prior children tended to report lower levels of internalizing symptoms at T2. Finally, women’s stress communication at T1 positively predicted men’s internalizing symptoms at T2. Regarding QoL, along with having prior children, higher common DC at T1 predicted higher QoL at T2 for all participants.

### Actor and Partner Effects of Change in DC on Change in Individual Adjustment

The univariate LCS models presented above emphasize that parity affects women’s and men’s change scores differently over time; therefore, this variable was left estimable in all models. The remaining parameters (i.e., the actor and partner effects between each change score and the effects of the remaining covariates on the change scores) were fixed to be equal across women and men. The models yielded a reasonably good fit (internalizing symptoms: χ^2^ = 157.52, *df* = 100, *p* < 0.001; RMSEA = 0.044; SRMR = 0.070; CFI = 0.971; QoL: χ^2^ = 141.95, *df* = 100, *p* = 0.004; RMSEA = 0.037; SRMR = 0.064; CFI = 0.978) (Table 4). For women, higher decreases in common DC (μ_Δ_ = −0.15, *p* = 0.009; σ^2^_Δ_ = 0.46, *p* < 0.001) were associated with higher change scores for internalizing symptoms (μ_Δ_ = -0.28, *p* = 0.308; σ^2^_Δ_ = 7.33, *p* < 0.001) and lower change scores for QoL (μ_Δ_ = 1.03, *p* = 0.320; σ^2^_Δ_ = 93.80, *p* < 0.001). For men, higher decreases in common DC (μ_Δ_ = −0.16, *p* = 0.002; σ^2^_Δ_ = 0.34, *p* < 0.001) were associated with increases in internalizing symptoms (μ_Δ_ = −0.80, *p* = 0.002; σ^2^_Δ_ = 5.89, *p* < 0.001) and decreases in QoL (μ_Δ_ = −2.16, *p* = 0.042; σ^2^_Δ_ = 104.85, *p* < 0.001).

**TABLE 4:** Associations between changes in dyadic coping and changes in individual adjustment.

	**Women**				**Men**			
	**Change in IS**		**Change in QOL**		**Change in IS**		**Change in QOL**	
	***R*^2^ = 0.16**		***R*^2^ = 0.13**		***R*^2^ = 0.12**		***R*^2^ = 0.16**	
	***B (SE)***	***p***	***B (SE)***	***p***	***B (SE)***	***p***	***B (SE)***	***p***
**Women change**								
Stress communication	−0.32 (0.24)	0.183	0.88 (0.97)	0.367	0.17 (0.25)	0.498	−0.95 (0.97)	0.327
Positive dyadic coping	−0.16 (0.32)	0.633	1.74 (1.30)	0.179	−0.17 (0.33)	0.602	0.88 (1.30)	0.500
Negative dyadic coping	−0.28 (0.25)	0.276	1.43 (1.02)	0.159	0.33 (0.25)	0.190	−1.83 (1.02)	0.072
Common dyadic coping	−**1.09 (0.28)**	**<0.001**	**4.53 (1.12)**	**<0.001**	−0.27 (0.27)	0.332	−1.03 (1.14)	0.368
**Men change**								
Stress communication	0.17 (0.25)	0.498	−0.95 (0.97)	0.327	−0.32 (0.24)	0.183	0.88 (0.97)	0.367
Positive dyadic coping	−0.17 (0.33)	0.602	0.88 (1.30)	0.500	−0.16 (0.32)	0.633	1.74 (1.30)	0.179
Negative dyadic coping	0.33 (0.25)	0.190	−1.83 (1.02)	0.072	−0.28 (0.25)	0.276	1.43 (1.02)	0.159
Common dyadic coping	−0.27 (0.27)	0.332	−1.03 (1.14)	0.368	−**1.09 (0.28)**	**<0.001**	**4.53 (1.12)**	**<0.001**
Parity^a^	−**1.50 (0.44)**	**0.001**	1.58 (1.67)	0.345	0.06 (0.41)	0.881	**4.46 (1.75)**	**0.011**

*Unstandardized maximum likelihood estimates are described. Significant estimates are in bold. ^*a*^0 = primiparous couples; 1 = multiparous couples. IS, internalizing symptoms; QOL, quality of life.*

## Discussion

This longitudinal study extends previous research that recently applied the STM to the transition to parenthood by considering both the prenatal and postnatal periods as well as internalizing symptoms and QoL as indicators of individual adjustment. Other strengths of this study include the consideration of the couple as the unit of analysis, which made it possible to explore the partner effects (mutual impact) as well as the beneficial and prejudicial effects (differential impact) of DC behaviors within couples that may have otherwise been missed. The key messages of this study are that (a) multiparous couples tend to present better individual adjustment over the second trimester of pregnancy until 6 weeks postpartum; (b) couples engaged less in joint coping efforts over time; (c) the more the couples engaged in joint coping efforts during pregnancy, the more they perceived quality in their life after childbirth; (d) the decline observed in couples’ engagement in joint coping efforts over time was accompanied by increases in couples’ depressive and anxiety symptoms, as well as decreases in their QoL; (e) members of the couple benefit differently from stress communication (being somewhat prejudicial for men when enacted by women) and positive DC (being somewhat prejudicial for women when enacted by them) in terms of psychological symptoms. These findings will be discussed below.

First, contrary to what we had hypothesized (hypothesis 1a), although women’s average levels of internalizing symptoms tended to be lower at postpartum than during pregnancy, decreases over time were neither statistically nor clinically significant. Our findings showed a trend toward improved psychological adjustment among experienced versus first-time mothers, which is very similar to the pattern observed in previous studies (Dipietro et al., 2008; Figueiredo and Conde, 2011). Moreover, first-time mothers are likely to manifest more emotional adjustment difficulties than experienced mothers in the early postpartum period, as previously observed (Gameiro et al., 2009), which could explain the lack of emotional warmth and empathy from pregnancy to postpartum by first-time mothers when their partners communicated stress (i.e., not taking the partner’s stress seriously, engaging in withdrawal behaviors), because previous studies suggested a positive association between negative DC and psychological symptoms (Rottmann et al., 2015; Alves et al., 2018). However, from a clinical perspective, the low scores for internalizing symptoms at each time point and the low difference values from T1 to T2, as well as between primiparous and multiparous women at each time point, did not allow us to make valuable conclusions about a potential better psychological adjustment among multiparous women over time. Rather, the findings could be interpreted as an indication that, overall, women have coped well with this transition. This is particularly interesting, considering that around 30% of women reported a prior history of psychopathology, which may have influenced, at some point, their adjustment.

Although men’s levels of internalizing symptoms tend to remain stable over time (supporting hypothesis 1b), first-time fathers’ well-being in certain life domains tends to decrease over the midpregnancy and early postnatal period, whereas an opposite trajectory is observed for experienced fathers. However, we should note that experienced fathers seem to present lower QoL during pregnancy than first-time fathers but that first-time fathers reached multiparous’ levels of QoL when becoming parents. This pattern of results is inconsistent with the findings of Chen et al. (2019), which showed that experienced fathers perceived less QoL in the physical health and social relations domains than first-time fathers over the perinatal period. The fact that the authors have assessed specific dimensions rather than a global perception of QoL, comparing scores from early pregnancy to 1 year postpartum, may explain the discrepancies between findings. For instance, other studies did not find associations between parity and father’s QoL (Pilkington et al., 2015). In our study, a past experience of parenthood appears to be a protective factor for both partners’ QoL at 6 weeks postpartum. This pattern of results could be attributable to the changes associated with the first-time transition to parenthood (Cowan and Cowan, 2000), in which couples may present some initial adjustment difficulties (e.g., Epifanio et al., 2015). In contrast, the absence of the novelty element (Gameiro et al., 2009) and the presence of more realistic beliefs about parenthood (Sockol and Battle, 2015) may have contributed to multiparous couples’ better adjustment from pregnancy to early postpartum.

Regardless of whether they were expecting a first or subsequent child, couples engaged less in common DC over time. As the pregnancy progresses, women experience several physical changes that, along with family and household responsibilities, may gradually contribute to intracouple imbalances regarding the provision of support. That is, in line with the predominant mother-centered medical care of this phase, men are likely to become more active in the couple’s relationship (Darwin et al., 2017), requesting less support (Levy-Shiff, 1999), than women. Indeed, we observed that men disclose less stress to their partners over time, and it is possible that this type of protective role toward women (Cowan and Cowan, 2000; Darwin et al., 2017) explains both their partners’ lower engagement in shared coping efforts (i.e., mutual efforts to cope with stress together are expected when both partners share stress). Less time spent together, tiredness due to lack of sleep, and decreases in intimacy, which are often observed after childbirth (St John et al., 2005; Delicate et al., 2018), could also explain our findings.

Regarding the long-term effects of DC on partners’ adjustment, our results confirmed only partially our hypotheses. Partners’ psychosocial adjustment was predicted by only three DC dimensions (common DC, positive DC, and stress communication) and not always in the expected direction. The finding that both partners have higher QoL when they actively participate in the coping process jointly supports the conceptualization of the transition to parenthood as a shared experience. Handling pregnancy concerns in a more or less symmetrical way (e.g., mutual efforts to calm one another’s pregnancy-related worries and uncertainties) may prevent both partners from feeling overwhelmed in the long term. Interestingly, although only marginally significant associations were found, the results indicated a trend toward lower QoL among couples who engaged more in positive DC. Contrary to the protective resource of common DC, engaging in supportive or delegated DC strategies to help each other cope with stress, while also facing significant changes and concerns during pregnancy (Canavarro, 2001; Kowlessar et al., 2015), can lead to increased overtiredness and then negatively impact both partners’ perception of their overall well-being. Surprisingly, contrary to previous studies in the field of partner support during the transition to parenthood (Pilkington et al., 2015), men’s provision of support did not predict women’s postpartum adjustment. These findings come to challenge the traditional role of fathers as the support provider and mothers as the care recipients (Darwin et al., 2017), highlighting that women *and* men benefitted mostly and equally from joint coping strategies regarding numerous dimensions of life. This adds on recent perinatal research suggesting that common DC is a key resource for partners’ relationship satisfaction (Molgora et al., 2019) and confidence in their parental role and against parenting stress (Alves et al., 2019). However, contrary to these studies, we found actor but not partner effects between common DC and partners’ adjustment (which did not confirm our hypothesis 2b). Although actor effects are, generally, stronger than partner effects in dyadic research (Kenny et al., 2006), it is interesting to note that, considering the results of Alves et al. (2019, 2020), partner effects of DC seem to be especially salient when DC is assessed *after* than before childbirth. The period soon after childbirth is likely to reinforce partner’s dependence in one another, as the birth of a baby affects both partners at the same time and as a unit. This rationale is sustained by the widely documented emotional interdependence between partners after childbirth (Goodman, 2004).

This can also explain why common DC at pregnancy was not found to be a significant predictor of internalizing symptoms, while the observed reduction in joint coping efforts over time was associated with increases in levels of psychological distress and decreases in QoL (supporting hypothesis 3). Over the course of pregnancy to the time after childbirth, stressors increasingly concern both partners, such as the changes in the relationship with one another, the need to share parenting responsibilities, and the need to negotiate new household routines (St John et al., 2005). The gradual reduction of adaptive strategies to jointly address these issues (e.g., spending time together and openly discussing one another’s concerns; Deave et al., 2008) could therefore make it difficult to adjust to the birth of a child.

Overall, it seems that a process of joint coping against stressors is a key resource for partners’ adjustment to the transition to parenthood to a larger extent than traditional forms of support. This rationale is supported by the result that the more women engaged in positive DC strategies to help their partners cope with stress during pregnancy, the more depressed and anxious they felt after childbirth. Although the direction of the association is inconsistent to what we have hypothesized (hypothesis 2), considering that they are the main source of support for men during pregnancy (Forsyth et al., 2011), engaging in DC strategies with their partners may have contributed to additional burdens at this sensitive time (Staneva et al., 2015) and therefore led to higher levels of psychopathological symptoms in the long term.

Finally, more communication of stress by women was found to increase men’s internalizing symptoms. The significant partner effect partially supports our hypothesis 2b, as the direction of the association is contrary to what we have hypothesized. However, this is in line with the mixed findings found in the literature, which has suggested that stress communication could be either considered an adaptive strategy (Vaske et al., 2015) or an unfavorable one when the negative content of the discussion takes a central role in the relationship (Meier et al., 2011). For instance, women reported communicating their stress more often than men during pregnancy (Alves et al., 2018; Molgora et al., 2019), which can be perceived as burdensome for men and thus contribute to higher levels of psychological distress. The reasons and mechanisms underlying the potential for certain DC strategies to contribute to feelings of burden and psychological distress should be addressed in further acceptability research with parents and health professionals. Qualitative research will also yield a more in-depth picture of the salience of the “we stress” experience of the transition to parenthood, as well as common DC when couples are managing stressors related to this period.

## Limitations of the Study

This study presents limitations, such as the high attrition over time, with lower retention rates for less educated couples, which limited the generalizability of the findings. However, the technique used for handling missing data (FIML) has been considered advantageous for handling a high proportion of missing data (Enders, 2010); accordingly, along with the inclusion of auxiliary variables, our findings can be interpreted with confidence. Nonetheless, future studies should elaborate on strategies for engaging and retaining individuals with low educational levels, as they represent a subgroup of couples that may have particular relationships with DC and internalizing symptoms. By assessing DC only with a self-report questionnaire, complex dyadic processes and interactions have been more difficult to capture. Studies with observational data and/or interviews with couples are warranted. Additionally, given the low internal consistency of the stress communication subscale for both women and men, with reliability values marginally below the acceptable threshold of 0.70, our findings should be interpreted with caution. Considering the low-risk sample of this study, its replication in different types of couples (e.g., couples facing high-risk pregnancies such as twins’ pregnancy) and considering normative potentially stress-inducing situations across this transition (e.g., proximity of delivery, return to work after parental leave) are recommended. Moreover, the achievement of larger and more diversified samples would facilitate the assessment of potential moderators of the associations between DC and adjustment, such as parity. On a related note, given the large number of predictors simultaneously considered in the analyses (which may have limited statistical power to detect theoretically meaningful associations), future studies, with desirable power to detect small to moderate effects, are needed to replicate these findings. Even though the rationale of this study assumed a causal path from DC to individual adjustment, we cannot exclude the possibility of alternative causal influences (e.g., couples in which one or both members experience psychological distress would probably engage less in joint coping efforts). Future studies with additional waves of data collection and cross-lagged paths are therefore warranted. Finally, we did not collect data about income and parental leave (in terms of use and length), which may have influenced couples’ adjustment to the birth of a child.

## Conclusion

The couples seemed to benefit more from a shared coping process than from specific strategies to assist their partners in managing prenatal stress. This finding informs us about a relevant dyadic process to foster among first-time and experienced parents and, importantly, suggests that approaches aimed at enhancing support processes for couples during the transition to parenthood need to be reconsidered. Rather than focusing excessively on increasing the support provided by one partner to the other, health professionals may consider helping couples to enhance ways to strengthen and maintain their engagement in joint coping efforts to handle common daily stressors across the transition to parenthood. Importantly, our findings suggest that such strategies should be promoted before, and fostered after, childbirth (e.g., by including a DC component in current pre and postpartum educational programs). While programs aimed to improve DC skills among couples already exist and whose efficacy has been acknowledged (e.g., Couples Coping Enhancement Training [CCET]; Bodenmann and Shantinath, 2004), our findings suggest that mental health professionals who intended to apply these interventions with couples in maternity care settings should be aware of both the similar (regarding common DC) and differential (regarding positive DC and stress communication) impacts of specific DC strategies within couples. Accounting for the mutual influences between partners and considering the sociocultural changes around the role of fathers (Cabrera et al., 2018), health professionals should address men’s needs along with those of the women.

## Data Availability Statement

The datasets presented in this article are not readily available due to ethical concerns about confidentiality. Requests to access the datasets should be directed to the corresponding author.

## Ethics Statement

The studies involving human participants were reviewed and approved by Research Ethics Committees of the Faculty of Psychology and Educational Sciences and Centro Hospitalar e Universitário de Coimbra, EPE. The patients/participants provided their written informed consent to participate in this study.

## Author Contributions

SA designed and implemented the study, conducted the data analyses, and wrote the manuscript. AF and MP assisted with the design of the study and the data analyses and collaborated with the writing and editing of the manuscript. MCC collaborated with the design of the study and writing of the manuscript. All authors contributed to the article and approved the submitted version.

## Conflict of Interest

The authors declare that the research was conducted in the absence of any commercial or financial relationships that could be construed as a potential conflict of interest.

## Abbreviations

APIM: actor–partner interdependence model
DC: dyadic coping
FIML: full information maximum likelihood
LCS: latent change score
QoL: quality of life
STM: systemic-transactional model
T1: second trimester of pregnancy
T2: 6 weeks postpartum.
